# The Decline of Basic Ophthalmology in General Medical Education: A Scoping Review and Recommended Potential Solutions

**DOI:** 10.1177/23821205241245635

**Published:** 2024-04-07

**Authors:** Jennifer Liao, Robin Redmon Wright, Gargi K Vora

**Affiliations:** 1 Department of Ophthalmology, The Robert Larner, M.D. College of Medicine, University of Vermont, Burlington, VT, USA; 2 Department of Behavioral Sciences and Education, 43324 Pennslyvania State University Harrisburg, Middletown, PA, USA; 3 Department of Ophthalmology, Yale School of Medicine, New Haven, CT, USA

**Keywords:** Ophthalmology education, primary care, undergraduate medical education, cognitive load theory, curriculum

## Abstract

**OBJECTIVE:**

This literature review aims to explore research and conceptual pieces on the state of ophthalmology education and suggest potential ways to address current challenges.

**METHODS:**

A search was conducted in PubMed, ERIC, Web of Science, and Google Scholar with combinations of the following search terms: “ophthalmology education,” “undergraduate medical education,” “medical student,” “United States,” and “Canada.” Eliminating irrelevant articles yielded 47 articles. Three were excluded because of region and focus, leaving 44. After examining the citations, we generated an additional 22 texts for review, totaling 66 articles.

**RESULTS:**

Four primary themes were identified: (1) challenges to ophthalmological education in the U.S. and Canada, (2) potential remedies for optimizing ophthalmology curriculum, (3) technology in ophthalmology education, and (4) innovative ophthalmology teaching approaches. Major challenges included the lack of a standardized curriculum and inadequate clinical exposure and skills training. A number of remedies were proposed, such as standardizing curriculum and furthering faculty involvement, utilizing technology as time-effective learning aids, and employing innovative teaching approaches such as service learning.

**CONCLUSION:**

In light of challenges in ophthalmology education, curriculum designers should consider Cognitive Load Theory (CLT) to assist students to remember meaningful exposures to ophthalmology knowledge and techniques. Based on CLT, we suggest two potential approaches to incorporating ophthalmology curriculum. The first is to embrace interdisciplinary collaborations and place ophthalmology knowledge in varied contexts to facilitate schema construction. The second is to incorporate ophthalmology diagnostics requirements into OSCEs and utilize simulation models for students to gradually increase the fidelity of tasks and devote cognitive resources fully to learning.

## Introduction

As the U.S. and Canadian population ages, demand for visual care will significantly rise in the next few decades.^1,2^ Consequently, it has become increasingly imperative that primary care physicians possess the basic skills to distinguish emergent eye findings from those that are chronic or benign. Major eye diseases that contribute to low vision and blindness such as cataract, glaucoma, and age-related macular degeneration increase in frequency with age.^3^ This is especially crucial for generalists who practice away from large academic centers in underserved communities with limited resources and inadequate access to readily available ophthalmology consultation. Nevertheless, there has been a continuous decline in ophthalmology education in medical school curriculum in the U.S. and Canada.^4^ Due to the rapid expansion of scientific information, Medical schools have increasingly focused on the core areas of medical education, rather than smaller specialties like ophthalmology.^5^ Furthermore, because the Liaison Committee on Medical Education does not specifically require ophthalmological training, the number of ophthalmology rotations and courses continued to dwindle in medical institutions across U.S. and Canada.^5^

Research shows that lack of a standard ophthalmology curriculum and clinical rotations has resulted in a negative impact on the basic ophthalmological skills of medical students and graduates. It was found that 70% of medical school graduates could not use an ophthalmoscope correctly, and most primary care residents in their study did not meet the ophthalmological skill standards set by the Association of University Professors of Ophthalmology (AUPO).^5^ Without basic ophthalmology evaluation skills, primary care physicians are unable to effectively discern chronic stable eye diseases from those that require immediate attention, possibly resulting in permanent damage and blindness that could otherwise be avoided while patients wait for specialist care. Potential remedies of ophthalmology curriculum should be explored to address these issues and improve ophthalmology education for medical trainees.

This scoping review explores the major research and conceptual pieces on the state of ophthalmology education in the U.S. and Canada to investigate potential ways to address current challenges in existing ophthalmology curriculum and better prioritize ophthalmology curriculum time in medical school curricula. Ophthalmology education encompasses a wide range of topics that are strongly applicable to other parts of medical education, including practical skills training, acquisition of scientific knowledge, clinical reasoning, decision making, as well as cultivation of empathy and interpersonal skills. Insights gained through this study will not only contribute to ophthalmology education but could also be extended to improve curriculum development in other areas of medical, health, and nursing education. Furthermore, this study provides a framework for curriculum development in areas beyond medicine to address gaps that require flexible solutions with a tight timeframe.

This study will begin with an overview of the methodology used. The next section will provide a discussion of the overarching themes found, followed by a comprehensive critique of current research. We frame our discussion of the literature through the lens of cognitive load theory (CLT), taking into account students’ cognitive architecture when devising recommendations for integrating ophthalmological learning tasks and activities into an already dense general curriculum.^4^ Finally, the conclusion will provide a summary of the main themes identified and recommendations for practice.

## Methods

**Figure 1.**
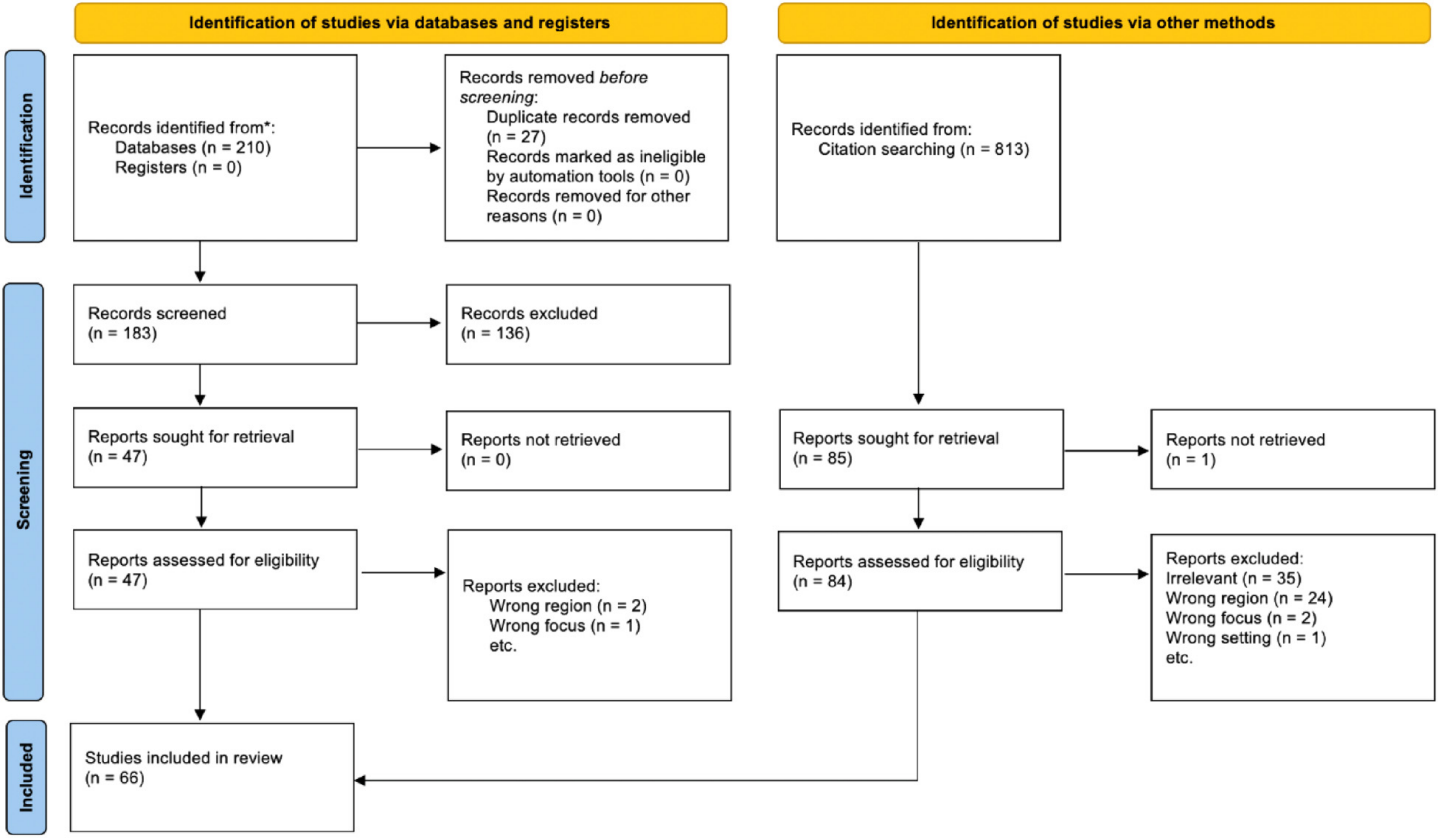
Prisma diagram.

We limit our study to the U.S. and Canada, where undergraduate medical education is completed after achieving a bachelor's degree and following a similar curriculum. The literature search was conducted on PubMed, ERIC, Web of Science, and Google Scholar, with various combinations of the following search terms: “ophthalmology education,” “undergraduate medical education,” “medical student,” “United States,” and “Canada,” including all articles from 2005 to 2021. The most recent search date was July 9, 2022. Two hundred and ten articles were generated from the search and screened for relevance to undergraduate ophthalmology education. Forty-seven articles passed the screening and were further reviewed; 3 of which are excluded because of region and focus, leaving 44 remaining articles. To cover articles not labeled by search terms, we have also examined citations of the above articles, which generated 22 extra texts, totaling 66 articles. See Figure 1 for the Preferred Reporting Items for Systematic Reviews and Meta-Analyses (PRISMA) flow diagram and Table 1 for literature details.

**Table 1.** The literature.

	Title	Author(s)	Type of article R = Research C = Conceptual	QN/QL/Mixed	Country	# of participants	Results and suggestion
Challenges to ophthalmology undergraduate education in the U.S. and Canada							
1	An analysis of undergraduate ophthalmology training in Canada	Noble et al (2009)	R	QN	Canada	386	First year residents who graduated from Canadian medical schools are surveyed. Most residents stated that they had little or no exposure to ophthalmology in medical school. Authors call for the development of a national, standardized curriculum to ensure that medical students acquire the necessary ophthalmology competency.^6^
2	Assessing the status of ophthalmology education 100 years after the Flexner report	Higginbotham et al (2010)	C		US		Authors reflected on the Flexner report in 1910 and urged for a similar reform focused on ophthalmology education, suggesting the establishment of new standards, incorporation of tools and education of all members of the specialty care team in ophthalmic core competencies.^7^
3	Current scope of online ophthalmology education and curriculum impact due to COVID-19	Lee et al (2021)	R	QN	US	50	Ophthalmology Directors of medical education (DMSEs) were surveyed about the scope of online curricula before and during the COVID-19 pandemic, as well as educators’ perceptions of online curricula and takeaways for future planning.^8^
4	Evaluation of Canadian undergraduate ophthalmology education at Western University	Li et al (2016)	R	QN	Canada	134	Authors evaluated the current level of ophthalmology knowledge and teaching curriculum for year 3 medical students at Western University medical school. Students demonstrated substantial deficiencies in ophthalmic knowledge and significant improvement after taking the newly designed lecture series.^9^
5	How prepared are medical students to diagnose and manage common ocular conditions	Esparaz et al (2014)	R	QN	US	194	Second-year and fourth-year medical students received a standardized 12 question quiz on diagnosing and managing ocular conditions. Authors found that both second-year and fourth year students have a significant gap in knowledge, and that students performed better on diagnosing than managing ocular conditions.^10^
6	Impact of coronavirus disease 2019 (COVID-19) on the ophthalmology training of Canadian medical students	Paco et al (2021)	R	Mixed	Canada	11	Authors surveyed Canadian undergraduate ophthalmology education leads and found three major themes: (1) Pre-clerkship lectures that used to be in person are becoming virtual. (2) Clinical skills training are impacted, some moving to online delivery. (3) Education leads call for development of online curricular resources.^11^
7	Medical students’ self-confidence in performing direct ophthalmoscopy in clinical training	Gupta et al (2006)	R	QN	Canada	208	Medical students were surveyed about their self confidence in performing direct ophthalmoscopy. A large proportion of students reported a lack of confidence in various aspects of their ophthalmoscopy skills. Most students were interested in additional training.^12^
8	Needs assessment of ophthalmology education for primary care physicians in training: comparison with the International Council of Ophthalmology recommendations	Chan et al (2017)	R	QN	Canada	162	A cross-sectional survey of family medicine residents showed that the average hour of ophthalmology training received by the residents meets the International Council of Ophthalmology suggestions, but most residents appear to be uncomfortable in managing common ophthalmic conditions.^13^
9	Ophthalmology in the medical school curriculum: reestablishing value and effecting change	Mottow-Lippa, Linda. (2009)	C		US		Authors described the efforts of the AUPO medical education Task Force to reintegrate ophthalmology into medical school curriculum, creating comprehensive recommendation on ophthalmology core competencies.^14^
10	Status of Canadian undergraduate medical education in ophthalmology	Gostimir et al (2018)	R	QN	Canada	14	Canadian medical school ophthalmology program directors were surveyed and only 5 of 14 schools were found to have a mandatory clerkship less than two weeks in length, and only 20% of the schools used a core curriculum based on ICO guidelines. However, all schools provide extracurricular exposure to ophthalmology through research opportunities and interest groups.^15^
11	Student perceptions of the ophthalmology curriculum in medical school	Cobbs et al (2018)	R	QN	US	166	Medical students at New York University School of Medicine (NYUSOM) are surveyed about their confidence with ophthalmology skills and level of satisfaction with the current curriculum without mandatory ophthalmology rotation. It was found that the majority of students found current ophthalmology training to be insufficient and lack confidence particularly in dilated eye and direct ophthalmoscope exams. Having more hours of training and having taken the ophthalmology elective were found to be associated with greater confidence in ophthalmology skills.^16^
12	The State of ophthalmology medical student education in the United States and Canada, 2012 through 2013	Shah et al (2014)	R	QN	US, Canada	135 (AUPO medical schools)	All AUPO member institutions in the US and Canada were surveyed to characterized their preclinical, clinical and extracurricular exposure to ophthalmology in 2012–2013. Most institutions were found to have some sort of preclinical ophthalmology experience and more variable clinical exposures, with the overall rate of required clinical rotations declining.^4^
13	The State of Ophthalmology medical student education in the United States: an update	Moxon et al (2021)	R	QN	US, Canada	117 (AUPO institutions)	117 AUPO institutions were surveyed to characterized their preclinical, clinical and extracurricular exposure to ophthalmology in 2020. Decreasing curricular time for ophthalmology has plateaued after the competence of graduating medical students had already been compromised, and ophthalmology teaching has shifted to preclinical years, in addition to extracurricular nontraditional teaching.^17^
14	Undergraduate ophthalmology education in Canadian medical schools: a cross sectional survey	Mah et al (2021)	R	QN	Canada	14	All Canadian medical schools were surveyed about their ophthalmology curricula. It was found that 13 out of 14 schools has preclerkship ophthalmology teachings while 7 offers an optional or mandatory ophthalmology rotation. Authors advocated for a required ophthalmology rotation in all schools.^18^
Standardizing curriculum and increasing faculty involvement							
15	A proposal to improve ophthalmic education in medical schools	Albert et al (2014)	C		US		Authors discussed the core knowledge of ophthalmology, causes for decline of ophthalmic education, and advocate the approach of establishing competency based standards in ophthalmology education.^19^
16	An international strategic plan to preserve and restore vision: four curricula of ophthalmic education	Tso et al (2007)	C		US		Authors highlighted the four international curricula of ophthalmic education developed by the Task Forces of the International Council of Ophthalmology in 2006.^20^
17	Evaluation of ophthalmology clerkships across teaching sites at the University of British Columbia	Nathoo et al (2019)	R	QN	Canada	207	Authors evaluated the ophthalmology clerkship program at the U of British Columbia, finding that difference in clinical experiences among clerkship sites did not develop into differences in learning as long as there is clearly outlined purpose, inputs, and activities.^21^
18	Medical student education	Lippa, LM (2006)	C		US		Authors discussed the medical student education gap in ophthalmology and call for curricular reform at both local curricular level and medical education circles of organized medicine level.^22^
19	Medical student education in ophthalmology: crisis and opportunity	Quillen et al (2005)	C		US		Authors discussed the current state of ophthalmology education and means to make positive impact, including prioritizing critical areas of ophthalmology learning, advocating formal ophthalmology student education programs, integrating ophthalmology to interdisciplinary medical curriculum, and developing innovative programs for medical students.^5^
20	Ophthalmology objectives for medical students: revisiting what every graduating medical students should know	Graubart et al (2018)	C		US		Authors proposed the adoption of a list of modified ophthalmology-related objectives for medical students.^23^
21	Refocusing ophthalmic education	Bellan, Lorne (2009)	C		Canada		Current deteriorating state of ophthalmology education was discussed and methods of redistributing curriculum time and seeking out new opportunities suggested.^24^
Utilizing technology in ophthalmology education							
22	A novel preclinical course in ophthalmology and ophthalmic virtual surgery	Yong et al (2012)	R	QN	US	23	Authors designed an ophthalmology elective with 3 parts: (1) web-based didactics, (2) vision screening in local clinic, (3) training with virtual eye surgery simulator. Students found the virtual surgery session a positive experience and were interested in having more experience with the simulator. Authors suggested that the learning modalities allows elective to hold a sustainable spot in curriculum without needing to depend on the availability of instructors.^25^
23	A self-directed preclinical course in ophthalmic surgery	Wu et al (2015)	R	Mixed	US	22	Authors created a preclinical course for ophthalmic surgery with 3 components: (1) web-based didactic modules, (2) 3-h interactive surgery simulation session, (3) 2 shadow experiences. Students were found to have increased knowledge in ophthalmic surgery after the course. Authors suggested similar flexible electives can help students explore interests and career choices.^26^
24	A virtual COVID-19 ophthalmology rotation	Wendt et al (2021)	C		US		Discussed the impact of COVID-19 on undergraduate ophthalmology education, conducted literature review on innovative learning strategies, and described a novel neuro-ophthalmology elective with a virtual curriculum that includes elements such as morning report, research, grand rounds, virtual patient encounters and an oral examination.^27^
25	Comparing Eyesi virtual reality simulator and traditional teaching methods for direct ophthalmoscopy: students’ perspectives at Indiana University School of Medicine	Tso et al (2021)	R	QN	US	31	While all medical students at University of Indiana completed a small group session for direct ophthalmoscopy in their first two years, students who enrolled in ophthalmology electives were invited for an additional session with the Eyesi Virtual Reality Simulator. Students’ confidence in direct ophthalmoscopy is significantly higher after they completed the Eyesi Simulator session, and 80% of the students felt that the Eyesi simulator was superior to traditional small group learning of direct ophthalmoscopy.^28^
26	Comparison of smartphone ophthalmoscopy versus conventional direct ophthalmoscopy as a teaching tool for medical students: the COSMOS study	Kim et al (2019)	R	QN	US	101	Medical students were randomized to start a training session with smartphone ophthalmoscope versus direct ophthalmoscope (DO) and switched to the other instrument in a second session. Significantly more students were able to visualize optic nerves with a smartphone ophthalmoscope compared to a DO. Students also reported higher confidence, preference, and higher ability visualizing the optic nerve with the smartphone ophthalmoscope.^29^
27	Development of an interactive anatomical three dimensional eye model	Allen et al (2014)	C		Canada		A digital, interactive, 3D virtual eye model was developed based on the principles of Cognitive Load theory (CLT).^30^
28	Multimedia learning tools for teaching undergraduate ophthalmology: results of a randomized clinical study	Steedman et al (2012)	R	QN	Canada	25	Authors evaluate the effectiveness of a novel multimedia learning tool (MMLT) for teaching a method of approaching common ophthalmologic presentations and found that students learning using an MMLT has higher knowledge retention and spent less time reviewing education content compared to students who read a textbook excerpt.^31^
29	The Future of medical education in ophthalmology	Sharma et al (2016)	C		Canada		Three macro trends in ophthalmology were discussed, including the exponential rise in smartphone trend, the development of massive online open access courses, and digitization of medical education.^32^
30	Assessing ophthalmoscopy skills	Afshar et al (2010)	R	QN	US	33	Authors reported the identification of optic nerve photos as an objective approach to assess students’ ability to visualize the optic nerve while conducting ophthalmoscopy.^33^
31	Assessment of direct ophthalmoscopy teaching using plastic canisters	Swanson et al (2011)	R	QN	US		A physical examination module using plastic canisters was developed to teach direct ophthalmoscopy to medical students and was found to be effective in developing their discernment skills.^34^
32	Evaluating medical students’ proficiency with a handheld ophthalmoscope	Gilmour et al (2017)	R	QN	US	33	Fundus photos from standardized patients (SPs) were obtained and place into a grid, and medical students were asked to examine SPs with a ophthalmoscope and match the patient to his/her fundus photo in a grid. Only 10 out of 33 students were able to correctly match the SP's eye to the photo and overall confidence was low. Authors proposed this method as a way to test students’ proficiency of visualizing the ocular fundus without the assistance of an instructor.^35^
33	Evaluation of an online Peer fundus photograph matching program in teaching direct ophthalmoscopy to medical students	Kwok et al (2017)	R	QN	Canada	95	Students were divided into experimental (n = 63) and control group (n = 32). Those in the experimental group practiced ophthalmoscopy with each other using an online peer fundus photograph matching exercise while the control group did not participate. All students participated in an ophthalmoscopy competition and the experimental group was more accurate in matching fundus photos compared with the control group. It was concluded that matching online fundus photos appear to increase the skill and confidence levels of medical students in ophthalmoscopy.^36^
34	Medical student ophthalmic knowledge proficiency after completing a clinical elective or an online course	Abou-Hanna et al (2020)	R	QN	US	79	Fourth year medical students voluntarily enrolled in one of two courses: the first is an online course titled “The Eyes Have It” (TEHI), and the second includes both TEHI and a two-week clinical component. Students completed the same written exam and a survey regarding confidence in managing ophthalmic conditions at the end of both courses. It was found that the online group scored only slightly inferior to the Clinical + Online group, suggesting that an online course may provide a sufficient knowledge base for those not intending to specialize in ophthalmology.^37^
35	Ophthalmology education in COVID-19: a remote elective for medical students	DeVaro et al (2020)	R	QN	US	18	A 4 week remote ophthalmology elective is designed for rising MS3 s and MS4 s at Emory University. The curriculum consists of online lectures, interactive activities, student presentations, case-based discussions and optional telehealth observations. After the course, students were tested with United States Medical Licensing Examination World (UWorld) test bank questions and were found to perform significantly higher than average UWorld question bank users.^38^
36	Perspectives on virtual ophthalmology education among Canadian medical students	He et al (2020)	R	QN	Canada	19	Medskl.com implemented an ophthalmology online learning program for medical students consisting of 4 synchronous webinars and 6 asynchronous video modules. Medical students who completed at least one webinar or module completed a survey about perceived efficacy. Students reported the insufficiency of medical school ophthalmology curriculum and that the access to virtual learning improved equitability and diversity in medical education.^39^
37	Teaching ophthalmoscopy to medical students	Mackay et al (2015)	C		US		Authors reviewed innovative methods of teaching ophthalmoscopy to medical students, including service learning and fundus photographs.^77^
38	Teaching ophthalmoscopy to medical students (the ToTeMS Study)	Kelly et al (2014)	R	QN	US	119	Medical students received training in ophthalmoscopy using simulators and human volunteers. Students were randomized to receive or not receive fundus photograph interpretation training. Students were found to prefer fundus photographs for learning and examining the ocular fundus, and identification of fundus features was also more accurate for fundus photos rather than direct ophthalmoscopy.^41^
39	Teaching ophthalmoscopy to medical students (TOTEMSII): a one year retention study	Mackay et al (2014)	R	QN	US	107	Follow up with the previous TOTEMS study to determine if differences between using ocular photos and direct ophthalmoscopy persists over time. It was found that one year after training, students still preferred using fundus photographs to using direct ophthalmoscopy and continued to be more accurate using photographs.^40^
40	The use of peer optic nerve photographs for teaching direct ophthalmoscopy	Milani et al (2013)	R	QN	US	131	Students were divided into experimental and control groups. Those in the experimental group had their optic nerve photo taken and received a photograph belonging to 1 of their peers. Students were given 3 days to identify the student matching the respective photograph. Authors found that more students in the experimental group showed improvement in direct ophthalmoscopy skills compared to those in the control group.^42^
Innovative ophthalmology teaching approaches							
41	A Novel 3 year longitudinal pilot study of medical students’ acquisition and retention of screening eye examination skills	Mottow-Lippa et al (2006)	R	QN	US	96	Authors assessed medical school students’ ability to perform and retain ophthalmic screening skills from second to fourth year. It was found that student's screening skills significantly deteriorated during third year clerkships and that reinforcing skills in the fourth year improved performance. Authors suggested that basic ophthalmology screening skills should be reinforced throughout the curricula to ensure competency.^43^
42	A pilot study on providing ophthalmic training to medical students while initiating a sustainable eye care effort for the underserved	Byrd et al (2014)	R	QN	US	12	Students participated in a service project named the community vision project. After 1 months, their direct ophthalmoscopy skills were compared with those of upperclassmen who completed all clerkships and internal medicine residents and found to be significantly higher. After one year, their ophthalmoscopy skill retention was tested and found to be significantly higher than nonparticipating classmates. Authors suggested that service learning offered an efficient model for incorporating ophthalmology education into undergraduate medical curriculum.^44^
43	A prospective study of the longitudinal effects of an embedded specialty curriculum on physical examination skills using an ophthalmology model	Mottow-Lippa et al (2009)	R	QN	US	84	Authors evaluated the effect of a longitudinal curriculum and student retention of ophthalmology screening skills across three years of medical school. It was found that skills decayed when not practiced, and additional training successfully reinforced performance, and authors suggested a reiterative training model for teaching physical examination skills.^43^
44	A randomized controlled study of art observation training to improve medical student ophthalmology skills	Gurwin et al (2017)	R	Mixed	US	36	Students were taught by professional art educators during 6 custom designed art observation sessions over 3 months. All completed pre- and post-testing, in which they described art works, retinal pathology images, and photographs of eye diseases. It was found that observational skills for art image, retinal images, and eye photographs significantly increased. Authors suggested art training as an effective method for improving ophthalmology observational skills.^45^
45	A required ophthalmology rotation: providing medical students with a foundation in eye-related diagnoses and anagement	Bowers et al (2021)	R	Mixed	US	67	The University of Pittsburgh Medical School (UPSOM) has a required 1-week long ophthalmology rotation as a part of the specialty care clerkship. The curriculum includes interactive case-based sessions, as well as handouts and homework that accompany clinical instructions. Student feedback are positive, highlighting the quality of teaching and received high clinical and exam scores, showing the clerkship to be engaging and effective.^46^
46	A systematic review of best practices in teaching ophthalmology to medical students	Succar et al (2016)	Lit Review				Literatures of best practices in ophthalmic education were reviewed and 3 main themes have evolved overtime: (1) The focus of ophthalmology education has shifted from what to teach students to how to teach; (2) Medical education has shifted from teacher-centered to learner-centered; 3) Medical education has moved from the apprentice model to competency-based model.^47^
47	Advancing ophthalmology medical student education: international insights and strategies for enhanced teaching	Succar et al (2019)	Lit Review				Researchers provided an update on new innovative ophthalmic teaching and learning practices literature.^48^
48	An interactive method for teaching anatomy of the human eye for medical students in ophthalmology clinical rotations	Kivell et al (2009)	C		US		Second year medical students enrolled in the ophthalmology rotation were taught eye and orbit anatomy through an interactive method. After review lectures on the anatomy of the eye, orbit and developmental anatomy of the eye, they were given a demonstration of the anatomy of the orbit on human cadaver. Students then dissects porcine eyes under low magnification with a microscope. Both student and faculty feedbacks showed high levels of satisfaction with this curriculum design.^49^
49	“Biology of the eye,” a novel multiformat translational elective for medical students	Marin et al (2019)	R	Mixed	US	17	Authors designed a 10-session ophthalmology elective for medical students, including lectures, journal reviews of key ophthalmology topics, and a dissection lab. Student surveys indicated an increase in knowledge as well as interest in research and clinical ophthalmology.^50^
50	Delivering mobile eye care to underserved communities while providing training in ophthalmology to medical students: experience of the Guerrilla Eye Service	Williams et al	R	QN	US	360	Patient records at the Guerrilla Eye Service, a medical student-led ophthalmology outreach service program, were examined and characterized. It was found that the program brought regular, comprehensive eye care to underserved communities in the Pittsburgh area, and provided referrals to university clinic for patients with advanced disease.^51^
51	Effectiveness of a 40-min ophthalmologic examination teaching session on medical student learning	Hoonpongsimanont et al (2015)	R	QN	US	30	Medical students interested in emergency medicine received a hands-on 40 min teaching session on slit lamp, tonometer and the ophthalmoscope. From the pre- and post-workshop questionnaires, it was observed that students’ confidence for those skills significantly increased.^52^
52	Evaluating the effectiveness of small-group training in teaching medical students integral clinical eye examination skills	Lee et al (2020)	R	QN	US	197	Second-year medical students enrolled in a small group training session consists of key components of the eye exam. Students’ direct ophthalmoscopy skills were assessed eight months later. It was found that students’ self-confidence increased in each aspects of the eye exam and they preferred the PanOptic ophthalmoscope over the traditional ophthalmoscope.^53^
53	Eye day for medical students: delivering ophthalmic undergraduate education through international collaboration	Tong et al (2016)	R	QN	Canada	28	A half day clinical and anatomy curriculum titled “Eye Day” was developed. Students read about eye anatomy and examinations and then the class was split into groups of 4 and rotated through seven 20-min stations designed to teach clinical skills and practice skills. Students enjoyed the experience and thought the session increased their ophthalmic knowledge. Eye Day was suggested as an efficient way to develop ophthalmic skills.^54^
54	Flipped ophthalmology classroom: a better way to teach medical students	Diel et al (2020)	R	QN	US	247	Authors designed an ophthalmology clerkship curriculum based on the flipped classroom model and implemented this curriculum in 2018. Medical students’ evaluation of the clerkship of the 2018 flipped classroom course was compared with that of the 2016 cohort and received higher ratings without changing test performances.^55^
55	Impact of a 1-day ophthalmology experience on medical students	Quillen et al (2006)	R	QN	US	121	Students participated in an 1 day ophthalmology curriculum consisting of a morning conference series and an afternoon case-based learning and eye examination skills session designed based on AUPO requirements. They also completed pre- and post-tests and it was found that the 1 day curriculum was effective in improving their ophthalmic knowledge and skills.^56^
56	Impact of near-peer education in a student run free ophthalmology clinic on medical student teaching skills	Chopra et al (2020)	R	Mixed	US	14	Fourth year medical students participated in near-peer education in the ophthalmology branch of the East Harlem Outreach Program (EHHOP). Participants reported increased confidence and comfort teaching ophthalmology concepts and skills. It was also found that senior students’ teaching skills gained did not align with junior students’ perception of teaching received. Authors concluded that the effectiveness of near-peer education in ophthalmology may be more effective later in training with a more solid foundation of knowledge.^57^
57	Innovations in ophthalmology education: a particular instantiation of general principles	Braun et al (2021)	C		US		Authors called for the reevaluation of medical education in the following aspects: reconsider how to address an ever-increasing amount of knowledge in limited time and whether lectures taught by lecturers of varying skill levels eventually benefit the students. They proposed medical educators to familiarize themselves with external learning resources such as mini lectures, and to take inspiration from humanities lectures and integrate an entertainment factor. Authors designed a course integrating the proposals and received positive qualitative response from students.^58^
58	Ophthalmic microsurgery lab for medical students: enhancing learner intrinsic motivation and comfort with microsurgery	Cole et al (2021)	R	QN	US	20	Medical students attended a lecture on corneal suturing via zoom, watched a video on operating microscopes, and attended microsurgery wet lab sessions. Students reported increased comfort with microsurgical skills, higher interest in ophthalmology, and high intrinsic motivation reflected by the mean Intrinsic Motivation Inventory (IMI) score.^59^
59	Ophthalmology service at student run free clinics: a national survey	Okaka et al (2021)	R	Mixed	US	14	Authors administered telephone survey to 14 Ophthalmology Student Run Free Clinics (SRFCs) across the US and summarized characteristics of current SRFCs, such as number of service hours, common diagnoses and challenges.^60^
60	Teaching clinical ophthalmology: medical student feedback on team case-based versus lecture format	Horne et al (2017)	R	Mixed	US	30	An ophthalmology elective utilizing the team-based learning approach was developed. Students participated in 4 sessions of TBL based on ophthalmology topics. They were found to show strong preference for team based learning in clinical ophthalmology compared to traditional lecture format.^61^
61	The efficacy of residents as teachers in an ophthalmology module	Ryg et al (2015)	R	Mixed	US	92	Researchers designed a longitudinal residents as teachers program consisting of 2 h workshops, voluntary recording of the residents’ teaching in small groups, and students’ feedbacks. It was found that resident teachers who took this training were as effective as community faculty teachers, and students responded overwhelmingly positively to resident teaching and noted their effectiveness in facilitating conversations.^62^
62	The Flipped Classroom: An Innovative Approach to Medical Education in Ophthalmology	Alabiad et al (2020)	R	QN	US	401	Second year medical students participated in a 1 week ophthalmology course designed for primary care providers. The course employed the flipped classroom approach, in which lectures were condensed into short videos, self-assessment quizzes, small group discussions and large group case-based discussion. Most of the students enjoyed the flipped classroom experience and prefer it over live lectures, and final examination scores in the flipped classroom course were comparable to those in the traditional course.^63^
63	The ophthalmology mini elective gives vision to preclinical medical students	Mortensen et al (2020)	R	QN	US	28	First and second year medical students enrolled in an ophthalmology mini-elective that consists of 4 weekly 2 h sessions. Each session begins with a 30–45 min lecture and ends with 2 h hands on learning, including a wet lab experience. Student evaluation responses show a significant increase in interest, confidence with ophthalmic knowledge, and comfort performing fundamental skills.^64^
64	Twelve tips for teaching ophthalmology in the undergraduate curriculum	Chadha et al (2021)	C		US		The article offered 12 tips for further integrating ophthalmology into the undergraduate medical education both in and out of the curriculum, such as identifying guidelines, using simulation tools, creating OSCEs and elective opportunities, as well as service projects, social media and online resources.^65^
65	University of Toronto's redesigned ophthalmology curriculum and eye dissection lab	Felfeli et al (2021)	R	QN	Canada	1640	Student feedback evaluations before and after the introduction of the redesigned foundations ophthalmology curriculum were compared. Students who participated in the new curriculum also completed a questionnaire about the eye dissection lab regarding their satisfaction and a knowledge based test. Students’ ophthalmology curriculum rating was found to be higher after the adoption of the redesigned curriculum. Students were also found to have higher scores in the satisfaction questionnaire, knowledge based test, and interest in ophthalmology after the eye dissection lab.^66^
66	UR Well Eye Care: a model for medical student ophthalmology education and service in the community	MacLean et al (2014)	R	QN	US	148	Researchers examined charts of consecutive patients seen at UR Well eye care, a student run underserved clinic and found that students were able to have a broad clinical exposure as well as provide much-needed service to underserved populations.1

QN = quant; QL = qual; Mixed = mixed methods.

### Statistical analysis

Data from most articles were collected from surveys, but some also included program information from institutional websites, test scores, and patient data. Use of data was varied based on the type of research. For quantitative studies and the quantitative part of mixed method studies, authors primarily used statistical analyses such as paired t-test and ANOVA. The qualitative data of mixed method studies consisted of collected written feedback from participating students, faculties, and focus group discussion records.

## Results

We identified four primary themes: (1) Challenges to ophthalmological education around the world, (2) Standardizing curriculum with more faculty involvement in medical school administration, (3) Utilizing technology in ophthalmology education, and (4) Innovative ophthalmology teaching approaches. The first theme outlined challenges and issues with current educational practices and the last three focused on proposed remedies to those challenges.

### Challenges to ophthalmology education

Many studies evaluated the state of ophthalmological education and raised challenges with regard to the lack of standardization in ophthalmology curriculum. Shah et al^4^ pointed out that the Liaison Committee on Medical Education, the organization responsible for creating guidelines for curriculum and accrediting medical schools, has no guidelines with respect to ophthalmology education. While the International Council of Ophthalmology (ICO) has recommended ophthalmology guidelines, they are not mandatory and thus not typically followed by medical schools. It was found that only 20% of Canadian medical schools have core curricula that follow the ICO guideline.^15^

The lack of a standardized core curriculum may have caused current ophthalmology curricula content and length to vary across institutions, since ophthalmology curriculum is left entirely to the institutions without mandatory guidelines. Moxon et al^17^ surveyed U.S. and Canadian institutions and found that while 94.7% of medical institutions include some form of ophthalmology exposure, the content varies institution by institution, with 78.9% requiring preclinical coursework only, 13.7% requiring both preclinical and clinical coursework, and 7.4% offered clinical exposure only. Chan et al^13^ also investigated the adequacy of medical school ophthalmology teaching and stated that there was a wide variation between ophthalmology instruction time in different institutions in Canada. Ophthalmology curricula seem to remain inconsistent across the U.S. and Canada, which poses a significant challenge to ophthalmology education.

Furthermore, ophthalmological clinical exposure and skills training has been reported to be inadequate in multiple studies.^4,6,10,15–17^ Shah et al^4^ surveyed U.S. and Canadian medical institutions in 2012–2013 and found that only 18% of institutions had required clinical exposure to ophthalmology, a drastic decline from 68% of institutions in 2000. In 2018, the percentage of U.S. and Canadian schools requiring an ophthalmology clinical rotation has further declined to 16%. In addition, 20% of medical schools do not require any exposure to ophthalmology skills training unless sought by individual students. As a result, 70% of medical school graduates do not know how to use an ophthalmoscope properly, and primary care residency program directors reported that most of their residents do not meet AUPO standards.^17^ In addition, Esparaz et al^10^ discovered that students not only had a substantial gap in ophthalmology clinical knowledge, but they also performed particularly inadequately on managing ocular conditions compared to diagnosing. The lack of management skills and ability to recognize when to perform a referral to an ophthalmologist significantly impacts the quality of care delivered to primary care patients. Overall, these studies reveal a pervasive lack of clinical rotations and skills training dedicated to ophthalmology, contributing to the lack of practical ophthalmology skills for primary care residents.

### Remedies to challenges

While all these articles recognized the problem of a lack of ophthalmological training during medical school, most focused on possible remedies. These studies fell into three camps: standardization of curriculum and more faculty involvement, utilization of technology, and innovative teaching methods.

#### Standardization of curriculum and involvement of faculty administrators

One remedy suggested by multiple sources^4,10,15,18^ is to standardize the ophthalmology curriculum by adhering to existing guidelines and establishing a baseline for curriculum duration and clinical components. Shah et al^4^ emphasizes further standardization of ophthalmology curriculum in medical school by integrating previous attempts like the AUPO standardization of core competencies,^14^ and the ICO guidelines for required training.^67^ Gostimir et al^15^ also recommended greater adherence to the ICO guidelines. They pointed out that the ICO strongly recommends using teaching methods including didactic lectures with clinical demonstration, and that clinical exposures and operating room experiences should be mandatory. Having conducted a longitudinal study of student acquisition and retention of ophthalmic knowledge, Lippa et al^68^ discovered that current skills training in the second year is not sufficient for students to retain and apply ophthalmic examination skills unless it is supplemented with the ophthalmology clerkship. They also suggested that medical schools should incorporate more ophthalmology skills training throughout the four-year curricula to ensure competency for graduates. While Moxon et al^69^ did not directly advise an increase in curriculum time, they also questioned whether the current number of ophthalmology hours and clinical exposure in medical school are sufficient to produce core competencies.

In addition to standardizing requirements to include all core competencies, some research suggests that ophthalmology faculty should be encouraged to become more involved in administration and to establish ties between the ophthalmology department and other departments as well as general medical education. Shah et al^4^ pointed out that ophthalmology departments tend to disengage themselves from medical student education and suggested increasing ophthalmology faculty participation in administrative roles may result in increased influence and awareness of the importance of ophthalmology training. Moxon et al^17^ suggested appointing a dedicated Director of Medical Student Education (DMSE) position for ophthalmology departments. They argued that institutions with a DMSE offer more clinical coursework (11.9 h vs 10.8 h) and have higher rates of faculty engagement. Gostimir et al^15^ recommended more evidence-based teaching that pairs ophthalmology education with other related fields. They suggested that a closer connection with medical school administration and educational practice will increase the influence of ophthalmology and likely lead to increased attention and curriculum time.

#### Utilizing technology in ophthalmology education

Multimedia technologies can be leveraged to improve students’ knowledge acquisition and self-regulated learning experiences in ophthalmology. Allen et al^30^ made use of cognitive load theory to develop a virtual interactive three-dimensional eye model, aiming to reduce cognitive load for learners and improve their learning of ocular anatomy. Steedman et al^31^ designed and evaluated multimedia tools in the form of presentations consisting of audio, video, text, and visual images. The authors noted that students assigned to multimedia tools received higher scores on questionnaires and spent 72% less time reviewing the educational content compared to those reading a textbook excerpt of the same content. Furthermore, prerecorded mini-lectures have become a new trend and a part of student-centered educational approaches. Research has shown that this method of dividing information into “bite-sized” materials is beneficial for students.^70^ Multiple studies utilized short, prerecorded videos in their ophthalmology teaching approaches and received higher student satisfaction, sense of involvement, and preference over traditional lecture format of teaching.^55,63^

The development of multimedia gave rise to virtual ophthalmology courses, which became even more essential and widespread during the COVID-19 pandemic. Lee et al surveyed all Directors of Medical Student Educations (DMSEs) of AUPO and found that while 44% of the institutions had no online component in their courses prior to the COVID-19 pandemic, the majority (78.3%) of the institutions have increased their online components in response to the pandemic.^8^ Previous in-person clinical courses are successfully transitioned into virtual courses, and extracurricular activities into online formats, allowing students increased flexibility and easier access to materials.^8^ Studies have found that virtual courses effectively enhanced student knowledge and interest in ophthalmology.^37,38^ In particular, Abou-Hanna et al^37^ compared the performance of medical students enrolled only in an online course with those enrolled in the same online course and an additional clinical component. They found that the online group scored only slightly inferior in the ophthalmology knowledge test compared to the combined online and clinical group, and suggested that online courses may provide a sufficient knowledge base and exposure to those not intending to specialize in ophthalmology. In addition, He et al found that virtual learning can mitigate inequality in medical education and promote inclusion.^39^ Even after the lift of the COVID-19 onsite restrictions, the online courses and resources developed will continue to transform medical education and provide increased accessibility and value to medical students.

Ophthalmoscopy training is another area in which diverse forms of technology are often leveraged to improve student learning. Ocular fundus imaging technology has been promising in improving medical students’ visualization of the ocular fundus and providing a method for instructors to objectively evaluate ophthalmoscopy training. Kelly et al^41^ conducted a study in which medical students learned to examine the ocular fundus through direct ophthalmoscopy and fundus photographs. They discovered that students preferred fundus photographs and were also significantly more accurate in identifying fundus features through photographs than ophthalmoscope. One year after the previous study, students still performed better using fundus photographs compared to direct ophthalmoscopy.^40^ Furthermore, in scenarios in which ophthalmoscopy is required, instructors usually have difficulty determining how accurate a student visualizes the fundus. Afshar et al^33^ suggested that fundus photos provide an objective way to assess a student's ability with ophthalmoscopy. They recruited volunteer patients, took photographs of their optic nerve in one eye, and then had medical students examine each patient with ophthalmoscope and match the respective volunteer with their optic nerve photo. Evidence such as the improvement of students in identifying the correct photo through training and the superior performance of ophthalmology residents and fellows supports the validity of the method.^33^ As such, fundus imaging techniques have provided new insights into the visualization of ocular fundus and ophthalmoscopy evaluation, and can be further leveraged through software applications to improve learning.^36^

Other tools such as smartphone ophthalmoscopy and ophthalmoscopy simulators also benefit ophthalmoscopy skill acquisition. Recently, many smartphone ophthalmoscopes have been developed, which capture ocular fundus images using a smartphone camera and an adaptor.^29^ Kim et al^29^ discovered that students were able to visualize the optic nerve better with the smartphone ophthalmoscope compared to the direct ophthalmoscope, and students also reported a more positive experience and higher confidence using the smartphone ophthalmoscope. They suggested that smartphone ophthalmoscopy can be a supplement or an alternative to current ophthalmoscopy teaching. Many studies have also utilized the Eyesi virtual reality simulator to assist student learning.^25,26,28^ A study by Tso et al discovered that students are generally more comfortable and confident in performing direct ophthalmoscopy on a patient following an Eyesi simulator session compared to traditional teaching session.^28^ In addition, the Eyesi simulator is also found to be able to accurately evaluate and differentiate skill levels in ophthalmoscopy.^71^ Integrating these technologies into direct ophthalmoscopy learning will provide valuable tools for student skill acquisition and evaluation.

#### Innovative ophthalmology teaching

Approaches to innovative ophthalmology teaching include new methods such as the flipped classroom, service learning, and art observation. The flipped classroom is a learner-centered educational paradigm that promotes active learning at the learner's own pace.^72^ Instead of giving lectures in class and having students complete assignments after class, the flipped classroom technique ask students to study prerecorded lecture videos or other materials ahead of time, and then enter the classroom for discussions, small group exercises, and projects, thus flipping the classroom.^72^ Upon comparing the traditional classroom experience with the flipped classroom experience, Diel et al^55^ discovered that students found the flipped classroom teaching session more valuable, experienced increased interactions with faculty, and sensed a higher level of inclusion as a part of the team. It was also noted that most students enjoyed the flipped classroom experience more than traditional lectures and expressed interest in continuing to receive instructions in this approach.^63^ In some cases, the learner also spent less time in the flipped classroom lectures compared to the traditional classroom approach, while the knowledge assessment performance remains comparable to traditional lectures.^63^ In the future, the flipped classroom can be further leveraged to promote more active engagement and higher student satisfaction in ophthalmology learning.

Service learning is a rapidly growing area that not only allows students to practice clinical skills, but also cultivates emotional competency through exposure to diverse patient populations and encouragement of systems thinking, as well as providing much-needed service for the underserved. In 2018, there are 23 student-run health clinics offering ophthalmology service over the United States, with each one providing 5.15 h per month of services on average.^60^ Many studies investigated demographics of the patient population and efficacy of the service clinic model on skills acquisition.^1,44,51^ MacLean and Hindman^1^ conducted research on the University of Rochester Well Eye Care service project and found that patients served by participating students came from diverse backgrounds, including undocumented immigrants and individuals exempted from filing federal tax returns due to low income. Another example is a study by Williams et al^51^ who studied a service program at the Guerrilla Eye Service at the University of Pittsburgh. They noted that students provided referrals to a university clinic for patients with advanced disease so that they can receive free longitudinal care there. Similarly, Byrd et al^44^ developed a community vision project that recruited second year medical students from the University of New Mexico to serve in the mobile clinic in Albuquerque and rural areas of the state of New Mexico. After a month, the students’ knowledge was compared with that of peers and internal medicine residents. They found that participants’ median knowledge assessment scores were 48% higher than their classmates and 37% higher than those of internal medicine residents, suggesting that service learning is an efficient model for incorporating ophthalmic training in medical curriculum.

Emphasizing the importance of observation in ophthalmology examinations, Gurwin et al^45^ conducted a randomized control study of art observation training on improving medical student ophthalmology skills. They randomized 36 students into 1:1 art training and control groups. Students in the training group received instructions from professional art educators during six 1.5 h art observation sessions over a three month period. All students completed pre- and post-testing in which they described a work of art, retinal pathology images, and photos of eye diseases. They found that observational skills measured by descriptive training significantly increased in the training group compared to the control group and suggested art observation may improve clinical observational abilities. Such innovative projects allow medical students to develop observational skills beneficial to ophthalmology skills acquisition while exposing them to educative experiences not usually offered in traditional settings.

## Discussion

This review highlighted the lack of ophthalmology exposure and curriculum standardization in medical school and analyzed promising efforts in improving ophthalmology education. In this age of rapidly expanding knowledge and increasingly crowded medical curricula, medical students are often overburdened with information. Given the limited amount of time of medical school and continuous advances in medicine, it is difficult to incorporate every field comprehensively in the medical curriculum. Future incorporations of ophthalmology instruction and practice should base instructional strategies upon the learner's cognitive capacity, and thus consider the integration of cognitive learning theory into curriculum design.

The cognitive learning theory (CLT) is a theory aimed to develop instructional design strategies based on human cognitive architecture.^69^ CLT assumes that the human cognitive system has a limited working memory and unlimited long-term memory composed of cognitive schemas. According to Cramer et al,^73^ “intrinsic cognitive load describes the inherent complexity of the information being taught, whereas extraneous cognitive load describes excessive instructional convolution, which distracts from the learning goals.” The ideal is reached with *germane cognitive load*. Germane load refers to the ideal amount of instruction and task complexity for promoting learning. As learners mindfully combine simple ideas into complex schemas in long-term memory, they develop expertise. CLT holds that the human cognitive system has a limited working memory that can only hold 5–9 elements and actively process 2–4 elements simultaneously.^69^ However, medical students often encounter complicated learning situations involving multiple elements of complex information and are often overloaded on information and unable to process that information into long-term memory.^74^ Consideration for the realities of cognitive load placed on learners when designing ways to add ophthalmology training to expansive medical education content could be accomplished by using approaches such as the competency-based curriculum^39^ and scaffolding instruction.^73^

Out of all the articles on curriculum, only Allen et al^30^ and Steedman et al^31^ utilized principles of cognitive load theory for curriculum design. Allen et al^30^ implemented a 3D eye model and interactive learning modules, designed to use both the learner's auditory and visual channels through visual modeling and voice narration to reduce cognitive overload. Secondly, they removed extraneous details from materials and information within the modules was divided into small chunks to limit the burden placed on working memory. Thirdly, students can hover their mouse over the anatomical subcomponents in the software and see visible labels, utilizing the special contiguity effect by keeping labels close to visual representations of these components. Finally, they incorporated a personal and welcoming narrative voice, intending to create a “personalization effect” that has been shown to improve learning. Steedman et al^31^ designed a multi-media learning tool (MMLT) for teaching ophthalmological contents and evaluating its effectiveness. They have developed the MMLT format based on CLT studies that found students retain more information when topics are presented in multiple channels. Both articles utilized tenets of CLT to better optimize the working memory of medical students when learning about ophthalmology.

Based on what we learned through this review and careful considerations of CLT, we suggest two potential remedies to incorporate ophthalmology time-efficiently into the medical school curriculum. The first is to embrace interdisciplinary collaboration between ophthalmology and other specialties such as neurology, dermatology, and ENT. By holding classes together with these specialties on overlapping topics and skillsets, repetition can be avoided and instruction time saved without compromising content. Furthermore, collaborating with other disciplines allows students to put ophthalmology skills in varied medical settings and assists the formation of schemas in long-term memory. Instead of memorizing ophthalmological concepts separately, students can learn about how an eye disease manifests in the context of other specialties, thus constructing their understanding of ophthalmological concepts under the larger framework of medical education.

A second approach is to incorporate ophthalmology diagnostics requirements into objective structured clinical examinations (OSCEs). While most students know the relation between certain conditions and systemic illnesses, physical diagnostics skills competencies are rarely enforced. Utilizing a formal simulation machine or simple creative simulation models such as those proposed by Kylstra and Diaz^75^ in OSCE allows students to demonstrate ophthalmological skills and visualize conditions. In the context of CLT, learning tasks should start from a low-fidelity (decreased element interaction) environment and gradually increase to a high-fidelity environment.^76^ By having students start from reading textual explanations, moving on to simulation, and finally to conducting diagnostics on real patients, the fidelity of the tasks is gradually increased and intrinsic load is reduced, freeing up resources to be solely devoted to learning. Gaming technology can also be leveraged to create simulation applications for medical students and assist their visualization, while allowing a limited degree of autonomy in exploring ophthalmological conditions. Utilizing teaching tools such as simulation software and gaming technology, combined with mandatory hands-on examinations on real patients can help students to retain knowledge and improve learning.

With the ever-increasing amount of information medical students are required to consume, CLT should always be a consideration when designing or updating medical school curricula. Careful curriculum design incorporating CLT, combined with learning technologies and innovative teaching approaches such as service-learning, will enable students to learn, process, and remember meaningful exposures to ophthalmological information and techniques. In the future, it will be increasingly important to strive for germane load in curriculum design and continue to seek creative ways of teaching that enable future physicians to achieve their ophthalmology competencies.

While this scoping review explored a variety of research in diverse topics, there are some inherent limitations in the types of articles available and survey questions used in these studies, leading to challenges in accurately assessing the quality of current ophthalmology education and devising effective strategies.

Firstly, almost all of the studies examined are quantitative except for some mixed-method studies, which also tended to focus on the quantitative aspects. While they provide important information about ophthalmology education through Likert-scaled surveys and data analysis on test scores, qualitative studies about ophthalmology curriculum and extracurricular activities are needed to provide insight about the students’ and educators’ points of view. A lot of questions about the transition from content-based to competency-based curriculum remain unanswered, such as whether students feel that the competency-based curriculum prepared them better for residency, or whether they encountered any major setbacks with the curriculum. Educators’ experiences and what they consider to be important would also be helpful for future educators as the competency-based curriculum continues to expand. Service-based learning is another topic area with great potential for qualitative research. It would be interesting to explore what students felt they learned from service-learning projects in order to understand their experience from a holistic perspective. While the focus on quantitative studies is understandable since ophthalmology is a relatively technical specialty, qualitative aspects of ophthalmology education should also be addressed for students and educators to have enhanced experiences in the future.

In addition, most of the surveys cited consisted of close-ended questions such as Likert scaled or yes/no questions, which can lead to different interpretations of the questions from what the researchers originally intended and do not allow participants to clarify their answers. A combination of close-ended and open-ended questions would provide more insight on the thought process of the person answering the survey.

## Conclusion

This review highlighted the inadequacies of ophthalmology education in the medical school curricula, including the lack of standardization of ophthalmology curriculum and insufficient clinical exposure and skills training. It also reviewed promising efforts to improve undergraduate ophthalmology education, such as standardizing curriculum and encouraging faculty involvement, utilizing technology to improve student learning, and employing innovative teaching techniques. Based on the tenets of Cognitive Load Theory, we have suggested two additional approaches to improve ophthalmic learning, including interdisciplinary collaboration with related specialties to reduce instruction time, and incorporating ophthalmological diagnostics requirements into clinical examinations and utilizing simulation technology to practice for these exams. We hope that the integration of CLT in curriculum design can inspire innovative teaching approaches and further improve physician capabilities in ophthalmology.
